# Prevalence and risk factors for *Staphylococcus aureus* nasopharyngeal carriage during a PCV trial

**DOI:** 10.1186/s12879-017-2685-1

**Published:** 2017-08-25

**Authors:** Abdoulie Bojang, Lindsay Kendall, Effua Usuf, Uzochukwu Egere, Sarah Mulwa, Martin Antonio, Brian Greenwood, Philip C. Hill, Anna Roca

**Affiliations:** 1 Medical Research Council Unit, P. O. Box 273, Fajara, The Gambia; 2 Faculty of Infectious and Tropical Diseases, London School of Hygiene & Tropical Medicine, London, UK; 3 Centre for International Health, School of Medicine, University of Otago, Dunedin, New Zealand; 4 Faculty of Epidemiology and Population Health, London School of Hygiene and Tropical Medicine, London, UK

**Keywords:** *S. aureus*, PCV, Nasopharyngeal carriage, Seasonality, Risk factor, The Gambia

## Abstract

**Background:**

We conducted an ancillary study among individuals who had participated in a cluster-randomized PCV-7 trial in rural Gambia (some clusters were wholly-vaccinated while in others only young children had been vaccinated), to determine the prevalence and risk factors for *Staphylococcus aureus* nasopharyngeal carriage.

**Methods:**

Two hundred thirty-two children aged 5–10 years were recruited and followed from 4 to 20 months after vaccination started. We collected 1264 nasopharyngeal swabs (NPS). *S. aureus* was isolated following conventional microbiological methods. Risk factors for carriage were assessed by logistic regression.

**Results:**

Prevalence of *S. aureus* carriage was 25.9%. In the univariable analysis, prevalence of *S. aureus* carriage was higher among children living in villages wholly-vaccinated with PCV-7 [OR = 1.57 95%CI (1.14 to 2.15)] and children with least 1 year of education [OR = 1.44 95%CI (1.07 to 1.92)]. *S. aureus* carriage was also higher during the rainy season [OR = 1.59 95%CI (1.20 to 2.11)]. Carriage of *S. pneumoniae* did not have any effect on *S. aureus* carriage for any pneumococcal, vaccine-type (VT) or non-vaccine-type (NVT) carriage. Multivariate analysis showed that the higher prevalence of *S. aureus* observed among children living in villages wholly-vaccinated with PCV-7 occurred only during the rainy season OR 2.72 95%CI (1.61–4.60) and not in the dry season OR 1.28 95%CI (0.78–2.09).

**Conclusions:**

Prevalence of nasopharyngeal carriage of *S. aureus* among Gambian children increased during the rainy season among those children living in PCV-7 wholly vaccinated communities. However, carriage of *S. aureus* is not associated with carriage of *S. pneumoniae*.

**Trial registration:**

ISRCTN51695599. Registered August 04th 2006.

## Background

*Staphylococcus aureus* is one of the most common bacteria associated with neonatal sepsis [1–3] and child pneumonia [4, 5] in sub-Saharan Africa. It is also a leading cause of community and hospital-based skin and soft tissue infections [6]. *S. aureus* is a common colonizer of the upper respiratory tract [7]. This asymptomatic colonization is a necessary step on the pathway to disease [8] with nasopharyngeal carriage [9], nasal and rectal carriage [10] being linked to subsequent *S. aureus* disease.

Asymptomatic carriage of *S. aureus* varies extensively between populations, age groups, gender, vaccination with pneumococcal vaccines, socioeconomic factors and may also show seasonal variation. In the USA, Taiwan, Mexico, and Gabon nasopharyngeal carriage rates in the general populations among participants age 1–90 years were reported to be 30.4%, 24.1%, 37.1% and 29.0% respectively [11–14]. In Cape Verde, prevalence of nasal carriage of *S. aureus* was reported to be higher among hospitalized individuals and health care workers than in the general population [15]. Nasopharyngeal carriage was also found to be elevated in an urban setting [16] and among HIV infected individuals [17]. In The Gambia, prevalence of *S. aureus* nasopharyngeal carriage was highest at birth and decreased during the first few weeks of life [18], although prevalence remains high during infancy [19].

The introduction of pneumococcal conjugate vaccines (PCVs) alters the microbial flora in the nasopharynx [20, 21] with a substantial decrease of pneumococcal serotypes included in the vaccine (vaccine types or VT) [22], and an increase in other pneumococcal serotypes (non-vaccine types or NVT) [23–25]. Among PCV naive population, several studies have shown that colonization with *S. aureus* is inversely associated with *Streptococcus pneumoniae* colonization [19, 26–30]. In addition, in one study, *S. aureus* carriage increased post pneumococcal vaccination with the highest differences between vaccinated and non-vaccinated children being found at the age of 12 months [31]. Although the inverse association between colonization with these two bacteria has been reported worldwide, the exact mechanism is yet to be understood. Some reports showed that production of hydrogen peroxide by the pneumococcus is directly bactericidal to *S. aureus* [32] and the presence of the pneumococcal pilus directly induce a host immune response that is deleterious to *S. aureus* colonization [33]. Lebon and colleagues [34] could not explain this inverse association between *S. aureus* and *S. pneumoniae* by measuring anti-pneumococcal antibodies with *S. aureus* colonizing and anti-staphylococcal antibodies with *S. pneumoniae* colonizing among healthy children.

Many of the studies showing the inverse association between these two bacteria are either small or the statistical analysis does not adjust for potential confounders. In a recent report from The Gambia, we showed that the inverse association found in the crude analysis was explained by the different carriage profile during the first year of life, with *S. aureus* decreasing in prevalence from birth and *S. pneumoniae* increasing, both reaching a plateau at around 20 weeks of age [35]. In this study, when age and other potential confounders were considered in the adjusted analysis, there was no association between the two bacteria with any of the *S. pneumoniae* endpoints analysed (overall carriage, VT carriage and NVT carriage). A number of other studies found no association between *S. pneumoniae* and *S. aureus* in either carriage [35, 36] or disease [37, 38].

The aim of the analysis presented here is to assess the prevalence and risk factors of nasopharyngeal carriage of *S. aureus* among children (5–10 years of age) living in rural Gambia who participated in a PCV-7cluster randomized trial.

## Methods

### Trial design

The analysis presented here is ancillary to a large, single-blinded, cluster-randomized (by village), placebo-controlled trial of PCV-7 conducted to assess the impact of vaccination on pneumococcal nasopharyngeal carriage. Details of the study design, and the overall impact of vaccination have been described previously [20, 39]. In brief, 21 villages in rural Gambia were randomized to two arms. Three doses of PCV-7 were given to all children below 30 months of age at the start of vaccination (July 2006) and to all those born in the study villages during the follow-up period (until July 2008) irrespective of the trial arm because PCV had been shown to be effective in this age group in a previous trial conducted in the country [40]. Vaccination in older children and adults depended on trial arm. In wholly vaccinated communities, all inhabitants received PCV-7. In partly vaccinated villages, the older age groups received one dose of serogroup C Meningococcal Conjugate Vaccine. PCV-7 vaccine was introduced in The Gambia as part of the Expanded Programme of Immunization across the whole country in August 2009 and replaced by PCV-13 in June 2011 [18].

### Longitudinal study

Approximately, 5441 inhabitants lived in the study villages. Six hundred and thirty-six subjects above the age of 30 months at the start of the trial were randomly selected from the 21 study villages for participation in the longitudinal study. Selection of participants was proportional to the number of subjects in each village for the different age groups (2.5 years to less than 5 years, 5 to less than 15 years and 15 years and above [41]). All subjects aged 5–10 years who had at least one NPS collected were included in the ancillary study presented here (232 subjects). We selected this age group for two main reasons: (i) both prevalence of *S. pneumoniae* and *S. aureus* nasopharyngeal carriage are high and (ii) it discriminates children vaccinated with PCV-7 (wholly vaccinated villages) versus children not vaccinated with PCV-7 (partly vaccinated villages).

### Ethical approval

Parental consent was obtained for children who participated in the original study which was approved by the joint MRC/Gambia Government Ethics Committee and by the ethics committee of the London School of Hygiene & Tropical Medicine.

### Sample handling

NPS were collected monthly during the first 3–4 months of follow-up (starting in November 2006) and then every 3 months until June 2008 [41]. Samples collection was done as described previously [20] in accordance with a WHO protocol [42]. The posterior wall of the nasopharynx was swabbed using a sterile calcium alginate swab and immediately inoculated into vials containing 1 ml of skim milk-tryptone-glucose-glycerol (STGG) transport medium. These vials were then placed in a cold box before being transferred to the Medical Research Council Laboratories in Fajara (a distance of 90 km) within 8 h of collection. The vials were then vortexed for a minimum of 20 s before being stored at −70 °C until tested in batches.

### Laboratory methods

Frozen STGG containing NPS were initially thawed on ice and then vortexed briefly for a minimum of 20 s. 50 μl of thawed medium was then plated on Mannitol Salt Agar (MSA) [CM0085, Oxoid UK] plates, the inoculum streaked into four quadrants in order to semi quantitatively determine the bacterial load before being incubated aerobically at 37 °C for 48 h [39]. The plates were later examined for pale to golden yellow doomed shaped colonies 1-2 mm in diameter. A catalase test was performed on all suspected colonies. Catalase positive colonies were tested further using the Staphaurex® plus kit [OXR30950201, Oxoid UK], a rapid latex agglutination test for the identification of *S. aureus*. Positive isolates were confirmed *S. aureus.*

### Data management and statistical analysis

The primary aim of this analysis was to assess the prevalence of nasopharyngeal carriage of *S. aureus* and determine risk factors in the study population. The rainy season was defined as the period from June to October and the dry season from November to May.

Firstly, summary statistics [median and IQR for the quantitative variables and (n%) for categorical variables] were estimated within each variable group. The distributions of these variables were compared between groups (*S. aureus* carriers versus non-carriers) using the Wilcoxon rank-sum test or Chi-square/Fisher’s exact test.

Further, logistic regression analysis with subject as a random effect was applied to quantify the association of *S. aureus* carriage with all the co-variables, reporting odds ratios (OR) and their 95% confidence intervals (95%CI) while adjusting for potential confounders. Co-variables that showed association with *S. aureus* carriage in the univariate analysis (*p*-value ≤0.05 were included in the multivariate analysis. Several plausible interaction terms were tested in the multivariable analysis using likelihood ratio tests at α = 0.050. The selection of interaction terms to be tested was exploratory.

A multilevel, random intercept, logistic regression modelling technique was applied to account for the correlations of samples collected from the same subject over time and the clustering of subjects within the same village. However, likelihood ratio tests (though conservative) showed little evidence (*P*-value >0.1) of within village clustering of subjects. Hence the random intercept models were simplified by ignoring the within village clustering of subjects. There was little evidence of an autocorrelation structure and an equal (independent) correlation structure was assumed. Furthermore, robust variance estimator were tested, but gave very similar results to the model based standard errors.

All the analyses were conducted in Stata 12.1 (StataCorp. Texas, USA). Figures were done in R statistical programming software (R Core Team 2014). *P*-values <0.05 have been taken to indicate statistical significance.

## Results

A total of 232 children [median age 5.7 years (range 5–10)] took part in the study, with 131 (56.5%) of the participants being residents in the PCV-7 wholly vaccinated villages. One hundred and twenty three participants (53.0%) were male; Jolas were the most common ethnic group (71.1%). Overall, we collected 1264 NPS [median = 6 per participant; range 1–10]. Demographic and epidemiological characteristics of the study participants are shown in Table 1.

**Table 1:** Baseline characteristics of the study participants

Variables	N	%
N	232	-
Sex		
Male	123	53.0
Female	109	47.0
Median age (Range)	5.7 (5–10 yrs)	-
Samples collected per child Median (Range)	6.0 (1–10)	-
Overall carriage	232	25.9
Missing data	11	0.01
Group		
Wholly vaccinated	131	56.5
Partially vaccinated	101	43.5
Ethnicity		
Jola	165	71.1
Fula	11	4.7
Mandinka	46	19.8
Other	10	4.3
Attended school		
Yes	141	60.8
No	91	39.2
Smoker in house hold		
Yes	104	44.8
No	128	55.2
Education		
None	86	37.1
< 1 yr	42	18.1
> =1	104	44.8
Ability to read		
Yes	74	31.9
No	158	68.1
Ability to write		
Yes	61	26.3
No	171	73.7

Prevalence of *S. aureus* nasopharyngeal carriage was 25.9% in the study population. In the crude analysis, prevalence of *S. aureus* nasopharyngeal carriage was similar between genders and age groups (Table 2). Higher prevalence of *S. aureus* carriage was found among children from villages wholly vaccinated with PCV-7 [30.9% vs. 23.0% OR 1.57 95%CI (1.14 to 2.15)] and children who had attended school for at least 1 year [29.9% vs. 23.8% OR 1.42 95%CI (1.06 to 1.91)]. In addition, prevalence of *S. aureus* nasopharyngeal carriage was higher during the rainy season compared to the dry season [33.7% vs. 24.8% OR 1.59 95%CI (1.20 to 2.11)]. Prevalence of *S. aureus* carriage was similar between children who were carriers or non-carriers of *S. pneumoniae* (either for any pneumococcal carriage, VT carriage or NVT carriage) (Table 2).

**Table 2:** Univariable analysis for association of *S. aureus* carriage with covariables

Variables	*S. aureus* ^+^	%	*S. aureus* ^−^	%	Total	OR	95% CI
	n		n				
N	347	27.5	917	72.5	1264		
Age (years)						1.05	0.96, 1.15
Sex							
Male	194	29.5	464	70.5	658		
Female	153	25.3	453	74.7	606	0.81	0.59, 1.11
Group							
Partially vaccinated	221	30.9	495	69.1	716		
Wholly vaccinated	126	23.0	422	77.0	548	1.57	1.14, 2.15
Ethnicity							
Jola	229	26.2	646	73.8	875		
Fula	17	33.3	34	66.7	51	1.42	0.67, 3.02
Mandinka	81	29.2	196	70.8	277	1.15	0.78, 1.69
Other	20	32.8	41	67.2	61	1.51	0.74, 3.10
Attended school							
Student	266	28.3	673	71.7	939		
Non-student	81	23.3	244	76.7	325	0.83	0.60, 1.16
Smoker in household							
No	189	28.0	487	72.0	676		
Yes	158	26.9	430	73.1	588	1.01	0.76, 1.34
Education							
< 1 yr	120	23.8	384	76.2	504		
> =1 yr	227	29.9	533	70.1	760	1.42	1.06, 1.91
Ability to read							
No	160	24.7	488	75.3	648		
Yes	187	30.4	429	69.6	616	1.32	1.00, 1.74
Ability to write							
No	198	25.6	574	74.4	952		
Yes	149	30.3	343	69.7	492	1.25	0.93, 1.68
Season							
Dry	221	24.8	669	75.2	890		
Rainy	126	33.7	248	66.3	374	1.59	1.20, 2.11
Any pneumococci							
No	129	30.4	296	69.6	425		
Yes	218	26.0	621	74.0	839	0.81	0.61, 1.07
Vaccine type (VT)							
No	311	27.3	829	72.7	1140		
Yes	36	29.0	88	71.0	124	1.10	0.71, 1.72
Non-vaccine type (NVT)							
No	156	29.9	366	70.1	522		
Yes	191	25.7	551	74.3	742	0.81	0.62, 1.07

**Fig. 1:**
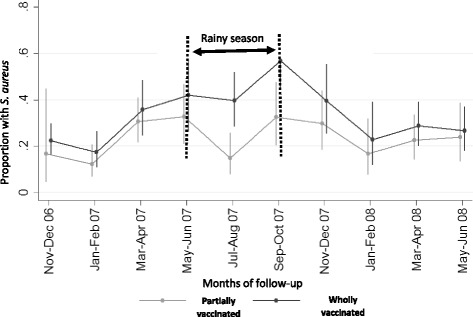
Bi-monthly prevalence of *S. aureus*

The multivariate analysis (including the analysis for interactions) showed that the higher prevalence of *S. aureus* nasopharyngeal carriage among children from PCV-7 wholly vaccinated compared to PCV-7 partially vaccinated communities only occurred during the rainy season [OR 2.72 95%CI (1.61 to 4.60)] and not in the dry season [OR:1.19 (0.82, 1.71)] (Fig. 1) (Table 3).

**Table 3:** Final multivariable analysis model showing factors associated with *S. aureus* carriage

Variable			n	OR	95% CI
Season	Dry	Partially vaccinated	399		
		Wholly vaccinated	491	1.19	0.82, 1.71
	Wet	Partially vaccinated	149		
		Wholly vaccinated	225	2.72	1.61, 4.60

## Discussion

This paper reports the prevalence of *S. aureus* nasopharyngeal carriage within the context of a PCV-7 cluster-randomized-trial in sub-Saharan Africa. Rural communities in The Gambia were randomized to higher or lower PCV-7 pressure (wholly versus partially vaccinated communities). Our findings support those of previous studies conducted in the country which showed that *S. aureus* carriage among children is high and not associated with *S. pneumoniae* carriage [35]. Nonetheless, in this study prevalence of *S. aureus* carriage was higher among children from communities with the highest PCV-7 pressure (wholly vaccinated communities) during the rainy but not the dry season.

Our results show that 25.9% of the Gambian children included in the study were carriers of *S. aureus* at any time point during the follow up period. It is difficult to make any valid comparison between this and other studies in the country since they differ either in the laboratory methods used, age, setting or exposure to PCV. In a similar study in the same setting among infants not exposed to PCV, prevalence of carriage was 30.9% [35]. In another study conducted in peri-urban Gambia among infants exposed to PCV-7, nasopharyngeal carriage of *S. aureus* was 33.6% whereas oropharyngeal carriage was 65.0% [43]. In Nigeria, *S. aureus* nasopharyngeal carriage among students aged 9–32 years attending various educational establishments was 56.4% [44].

Our study revealed that despite the introduction of PCV-7, no association between *S. aureus* and *S. pneumoniae* nasopharyngeal carriage was observed for any of the *S. pneumoniae* end points. These results are in line with previous data in the same villages before PCV introduction which showed no association between these two bacteria among infants [35]. Our trial had previously showed a strong herd effect of PCV-7 introduction in communities partly vaccinated with PCV-7 [20, 45] which turned into similar pneumococcal serotype distribution in the two trial arms. Therefore, the reasons for higher prevalence of *S. aureus* in PCV-7 wholly vaccinated communities during the rainy season are unclear. One possible explanation would be that risk factors of *S. aureus* carriage differ between study arms, and thus prevalence of carriage was already different between trial arms before PCV-7 introduction. However, this is unlikely due to the nature of the trial where clusters were randomly selected. It could also be a chance finding and we note that a number of variables were studied without a prior hypothesis. Further studies will be needed to explain these findings.

In our analysis, carriage of *S. aureus* follows a seasonal pattern which peaks during the rainy season unlike *S. pneumoniae* which peaks in the dry season [41]. In line with this seasonality of carriage, Chan and colleagues [46, 47] reported increased maternal *S. aureus* colonization (RR = 1.96, 95% CI 1.29 to 2.95) during the rainy season in Bangladesh and a study in Australia also reported an increase of *S. aureus* infection during the rainy season [48]. In our setting, infections with respiratory viruses such as RSV, influenza A, influenza B or adenoviruses are higher during the rainy season when humidity increases [49]. Initial colonization with respiratory viruses or decreased immune competence due to poor diet during the rainy season may have resulted in the increased transmission of *S. aureus* [50, 51]. However, we did not assess either viral infection or diet in study participants.

Our study was limited by a number of factors related to the design. First, we limited this ancillary analysis to children 5–10 years of age for two main reasons. Bacterial transmission among children tends to be higher than in adults. Also, in this age group children had only received PCV-7 if they live in the fully vaccinated villages which is not the case among younger children. On the other hand, our results on seasonality are based in a short time period. A longer follow up would have been more robust in establishing a seasonal pattern. The study only collected NPS for the detection *S. aureus*. Although prevalence of carriage would have likely been higher if the samples collected were oropharyngeal swabs, we do not expect different associations in the risk factors analysis.

## Conclusion

Our findings show that transmission of *S. aureus* in our setting is high which may explain the high burden of associated disease [49]. In addition, we did not find any association between *S. aureus* and *S. pneumoniae* nasopharyngeal carriage. The effect of PCV introduction on *S. aureus* transmission in rural Gambia will need to be further studied after the recent introduction of PCV-13 as part of the Expanded Programme of Immunization to determine if our findings are confirmed.

## Abbreviations

CI: Confidence interval
MRC: Medical Research Council
MSA: Mannitol Salt Agar
NPS: Nasopharyngeal swab
NVT: Non vaccine type
OR: Odds ratio
PCV (7/13): Pneumococcal conjugate vaccine (7/13) valent
RSV: Respiratory syncytial virus
*S. aureus*: *Staphylococcus aureus*
*S. pneumoniae*: *Streptococcus pneumoniae*
STGG: Skim milk-tryptone-glucose-glycerol
VT: Vaccine type
